# Antiproliferative Properties of Type I and Type II Interferon

**DOI:** 10.3390/ph3040994

**Published:** 2010-03-30

**Authors:** Joseph Bekisz, Samuel Baron, Corey Balinsky, Angel Morrow, Kathryn C. Zoon

**Affiliations:** National Institute of Allergy and Infectious Diseases, National Institutes of Health, Bethesda, MD 20892, USA

**Keywords:** interferon, apoptosis, antitumor, monocytes

## Abstract

The clinical possibilities of interferon (IFN) became apparent with early studies demonstrating that it was capable of inhibiting tumor cells in culture *and in vivo* using animal models. IFN gained the distinction of being the first recombinant cytokine to be licensed in the USA for the treatment of a malignancy in 1986, with the approval of IFN-α2a (Hoffman-La Roche) and IFN-α2b (Schering-Plough) for the treatment of Hairy Cell Leukemia. In addition to this application, other approved antitumor applications for IFN-α2a are AIDS-related Kaposi’s Sarcoma and Chronic Myelogenous Leukemia (CML) and other approved antitumor applications for IFN-α2b are Malignant Melanoma, Follicular Lymphoma, and AIDS-related Kapoisi’s Sarcoma. In the ensuing years, a considerable number of studies have been conducted to establish the mechanisms of the induction and action of IFN’s anti-tumor activity. These include identifying the role of Interferon Regulatory Factor 9 (IRF9) as a key factor in eliciting the antiproliferative effects of IFN-α as well as identifying genes induced by IFN that are involved in recognition of tumor cells. Recent studies also show that IFN-activated human monocytes can be used to achieve >95% eradication of select tumor cells. The signaling pathways by which IFN induces apoptosis can vary. IFN treatment induces the tumor suppressor gene p53, which plays a role in apoptosis for some tumors, but it is not essential for the apoptotic response. IFN-α also activates phosphatidylinositol 3-kinase (PI3K), which is associated with cell survival. Downstream of PI3K is the mammalian target of rapamycin (mTOR) which, in conjunction with PI3K, may act in signaling induced by growth factors after IFN treatment. This paper will explore the mechanisms by which IFN acts to elicit its antiproliferative effects and more closely examine the clinical applications for the anti-tumor potential of IFN.

## 1. Historical Perspective

In 1957 Isaacs and Lindenmann first described interferon (IFN) as an antiviral agent. [1] The antiproliferative effects of IFN were first described in 1962 by Paucker, who showed that a 24 hour exposure of L cells to either UV-irradiated Newcastle Disease Virus or to interferon led to a temporary decline in the growth of the cells. [2] This effect is seen in both malignant as well as non-malignant cells of many cell lineages with the degree of the effect varying greatly among cell lines. Shortly thereafter, the ability of IFN preparations to both slow the growth and inhibit cellular transformation ability of oncogenic viruses (e.g., Polyoma virus) was described. Oxman and others showed that the cellular transformation and neoantigen (SV40 T antigen) formation induced by oncogenic viruses like SV40 could be blocked by pre-treatment of cell cultures with IFN. Interestingly, this effect was seen when normal 3T3 cells were pre-treated with IFN and subsequently infected with SV40 but was not observed in a line of SV40-transformed 3T3 cells [3]. This IFN pre-treatment effect on oncogenic viruses was also seen in animals inoculated with polyoma virus as evidenced by delay in tumor appearance as well as a decrease in tumor size and number. Given the apparent necessity for pretreatment with IFN, results of work done with mice infected with Friend and Rauscher leukemia virus was unexpected. Interferon treatment of the mice post-infection was seen to reduce characteristics associated with leukemia (e.g. splenomegaly) with an increase in mouse survival. [4,5] In addition to IFN’s effect on Friend and Rauscher virus, it was shown to both inhibit both the growth of the Mouse Sarcoma Virus (MSV) as well as its ability to transform mouse embryo fibroblasts (MEFs). Indeed, it was shown that a continued treatment of cell cultures infected with MSV resulted in an inhibitory effect of MSV focus formation [5,6], and in 1970 Chany showed that when MEFs were exposed to IFN induced by Newcastle Disease Virus (NDV) for 200 passages the resulting cells lost properties of the cell population originally transformed by MSV. Unlike the ability of transformed cells to produce colonies in soft agar, the IFN-treated transformed cells no longer had this ability. In addition, cells treated with IFN lost their spindle shape and became epithelial. The changes were so significant that Chany gave the new, IFN-treated line the name MSV-IF^+^. In addition, Chany draws a parallel with his *in vitro* work and that of Gresser’s *in vivo* work in which Gresser showed that even highly purified mouse IFN can decrease the replication of some ascetic tumor lines induced by carcinogens [7,8]. 

Considerable work has been done on the effects of interferon on human malignancies. Work done by Gresser and colleagues which examined the ability of IFN to reverse the phenotype of transformed and tumorigenic cells to a more normal phenotype showed a partial reversion in human osteosarcoma cells (OHA) but no reversion in bladder carcinoma EJ cells after long-term IFN treatment. [9] A delay in mammary tumor development in female mice after receiving IFN led to work on the effects of IFN on human breast cancer xenografts implanted in athymic nude mice. Two of the three human tumors were sensitive to IFN-α [10,11]. Clinical trials were also done examining the effects of IFN treatment on malignant melanoma, multiple myeloma and acute granulocytic leukemia and chronic lymphatic leukemia, all showing some activity as marked by tumor regression or delay in tumor growth but the malignancy with the distinction of being the first licensed application of IFN-α was Hairy Cell Leukemia (HCL) which occurred in 1986. 

The fact that interferons can bring about long-term remissions in certain malignancies is well established however, the mechanism(s) by which this is achieved is a matter of continued study. In this review, we will explore the numerous aspects of IFN’s ability to inhibit tumor growth and the mechanisms that lead to growth inhibition and cell death. We will also discuss how human immune cells, e.g., monocytes, used in conjunction with IFN can enhance the anti-tumor effect. Given the pleiotropic nature of IFN, a description of some of the proteins expressed as a result of IFN and their possible role in the anti-tumor mechanism is presented. Finally, this review would not be complete without citing the anti-tumor clinical applications of those IFNs which have been licensed by the United States Food and Drug Administration.

## 2. Molecular Mechanisms of IFN Action

### 2.1. Cell cycle inhibition and antiproliferation

Interferon is known to affect different phases of the mitotic cycle in different cell systems with the most common effect being G1 arrest [12]. Eukaryotic cells are dependent on the sequential formation and activation of a series of serine/threonine protein kinases which are comprised of the regulatory component cyclin and a catalytic component known as cyclin-dependent kinase (Cdk) [13]. Work done by Asano *et al*. on the effect of IFN-α on cell cycle arrest of mouse macrophages showed that the Cdk inhibitors p19 and p21 were strongly up-regulated after treatment with IFN-α, and that that the binding of these inhibitors to the G1 cyclin/Cdk complex leads to reduction of its kinase activities and results in G1 arrest in the early phases of IFN treatment [14]. Interferon treatment also induces Cdk inhibitors p15 and p27 [15,16,17], resulting in cell-cycle arrest at the G1 phase. Interferon’s effect on cell growth inhibition was studied in three human lymphoid cell lines: Daudi, U-266 and H9, the latter of which is completely resistant to the antiproliferative effects of interferon. The Daudi and U-266 cells differ in that they arrest at different phases of the cell cycle, with Daudi cells accumulating in a G0-like state (with consequent inhibition of cyclins D3 and cdc25A) and the U-266 cells arresting subsequent to the G1 phase [18,19]. 

The transcription factor c-myc has been shown to be down regulated in response to type I interferon, resulting in cell-cycle arrest [20,21,22,23] and the induction of cell-cycle progression by c-myc is due in part to inhibition of translation by dephosphorylation of 4E-BP1 and activation of eIF4E and increased phosphorylation of the alpha subunit of eIF2α [24,25]. Activation of the IFN-inducible gene RIG-G also results in up regulation of Cdk inhibitors p21 and p27 [26]. Interferon inhibits phosphorylation of the tumor suppressor protein pRb. The unphosphorylated form binds the transcriptional activator E2F, resulting in cell cycle arrest at the G1 phase [27,28,29]. 

Other signaling pathways have been shown to be activated by IFN treatment. Binding of Type-I IFNs to the IFNAR complex induces phosphorylation of the vav proto oncogene, which in turn activates the GTPase Rac1, resulting in phosphorylation of p38 MAPK [30,31]. It is also activated by a wide array of stress responses, such as radiation and heat shock, and play roles in the signaling cascades that induce gene transcription for induction of cytokines, organize the actin skeleton, and regulate hepatocye growth [32,33]. 

**Figure 1:**
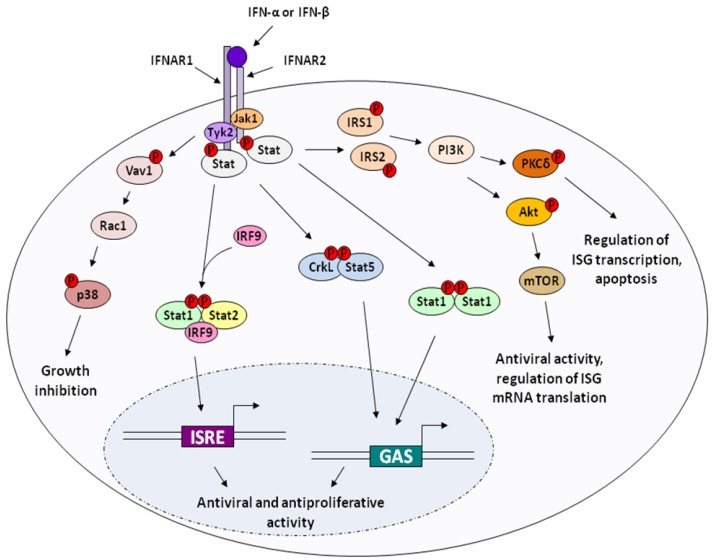
Type I IFN Signaling. Type-I IFNs bind to a heterodimer composed of the transmembrane receptor subunits IFNAR1 and IFNAR2, which results in activation of the receptor-associated kinases Jak1 and Tyk2. In the canonical signaling pathway, the cytoplasmic signal transducers and activator of transcription (Stat) proteins are recruited to the receptor docking sites, phosphorylated, and dimerize to form the active transcription factors ISGF3 (Interferon stimulated gene factor 3), composed of phosphorylated Stat 1 and Stat2, and IRF9 (p48/ISGF3γ), and AAF/GAF (alpha activation factor/gamma activation factor), which is composed of two phosphorylated subunits of Stat1. These induce transcription of hundreds of interferon-stimulated genes (ISGs). IFN treatment also leads to induction of other non-canonical signaling pathways, including those involving p38 MAPK, Akt, and Crk. Upstream signaling from the IFNR complex leads to p38 phosphorylation, which modulates IFN activity and leads to growth inhibition of cells. CrkL and CrkII are tyrosine phosphorylated by Tyk2 after IFN treatment, and CrkL can also form transcription factor complexes with phosphorylated Stat5. Signaling pathways downstream of PI3K, involving Akt and mTOR, or PKCδ, are also important in mediating the biological activities of IFN.

Stress-induced responses result in p38 MAPK-induced phosphorylation of Stat1 at S727, but this has not been shown to be the case for IFN-γ [34]. The precise mechanism by which this kinase induces IFN-induced gene transcription therefore remains elusive. It is a key mediator of inflammation as well as immune response and it regulates the expression of multiple cytokines and cellular receptors [35]. P38 MAP kinases also play an important role in cell differentiation and apoptosis and are thought to be important mediators of tumor progression [36,37]. 

Both Type-I and Type-II IFN treatment activates the Crk signaling pathway. CrkL and CrkII are tyrosine phosphorylated by Tyk2 after IFN treatment [38,39]. These proteins may interact with the GTPase Rap1, which antagonizes the Ras pathway and may lead to the IFN-induced antiproliferative effect on bone marrow hematopoietic progenitor cells [33,39]. In addition, CrkL also forms transcription factor complexes with activated Stat5 and binds to GAS sequences to induce gene transcription [33,40] (Figure 1).

### 2.2. Apoptosis and Cytotoxicity

Unlike antiproliferation, apoptosis is a form of programmed cell death (PCD). Its biological implications first being appreciated almost 40 years ago, apoptosis is now accepted both as a mechanism which can suppress tumorigenesis as well as being important in anti-neoplastic therapy. It is interesting to note that different cell types may have either pro- or anti-apoptotic reactions to IFN and that the apoptotic response to IFN treatment is not observed with many malignant cells. For example, more than ten years ago, cell lines which were established from moderately differentiated squamous cell carcinomas underwent cell death when exposed to IFN-α with no flow cytometric evidence of cell cycle arrest. In addition, transmission electron microscopy (TEM) of the cells revealed morphological changes associated with apoptosis (e.g., cellular shrinkage, condensation of nuclear chromatin) supporting an association between IFN-α and induction of apoptosis in these cells [41]. Conversely, in certain cell systems, IFN-α has been reported to actually protect certain cells from chemical- or glucocorticoid-induced apoptosis while IFN-γ can protect against p53-induced apoptosis [42]. Milner *et al*. found that in a panel of Burkitt’s lymphoma cell lines tested, all but Daudi cells were protected from apoptosis induced by ionomycin, which is a calcium ionophore known to stimulate rapid apoptosis in the presence of IFN [43]. One year prior to this, it was shown that B-chronic lymphocytic leukemia (B-CLL) cells which, while they undergo apoptosis in culture, are prevented from doing so when in the presence of IFN-α [44]. There are a few possible reasons for the disparity in IFN action as either inducing or protecting against apoptosis. It may be tumor-related, associated with the environment of the tumor or due to other cellular components and their secreted factors. The degree of cellular differentiation may also play a role, with certain cells being more likely to target genes that regulate apoptosis. 

The differentiation between inhibition of proliferation and apoptosis can be made using flow cytometry and transmission electron microscopy, and it is aided by the availability of agents known to induce apoptosis (e.g., corticosteroids, gamma irradiation). It has been shown that the two mechanisms are independent of each other and possibly indicative of each mechanism following a different pathway [18].

**Figure 2:**
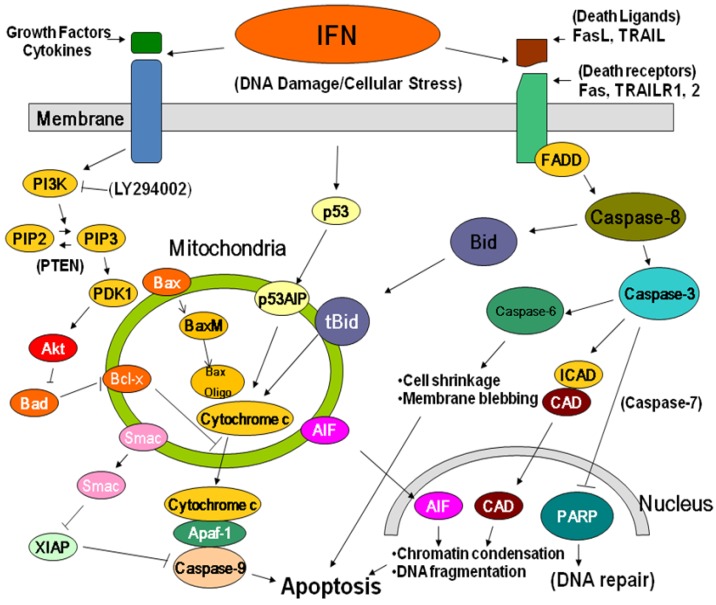
Apoptotic Signaling in Response to DNA Damage. The caspase cascade is initiated by chemical damage to DNA which stimulates Bid cleavage leading to permeability transition of the mitochondrial membrane. The mitochondria releases cytochrome c in response to apoptotic signals and serves to activate Apaf-1 with consequent activation of Caspase 9 and the remainder of the caspase cascade. These caspases transmit the apoptotic signal which eventually leads to cell death.

Treatment with IFN-α,β or γ results in the up regulation of pro-apoptotic proteins such as Fas, Fas-ligand (FasL) and TRAIL [45,46,47,48,49,50]. These proteins can interact with FADD (Fas associated death domain) or TRAIL-receptor proteins, resulting in initiation of apoptosis through activation of caspase-8 [51,52]. Interferon can also up regulate caspase-4 and caspase-8 in certain cell lines [53,54,55], and can activate the initiator caspases-8 and 9, as well as the effector caspase-3 (Figure 2). These proteins can result in increased sensitivity of cells to pro-apoptotic stimuli such as TNF-α.

In some cells, the tumor suppressor gene p53, which functions to initiate cycle-arrest or apoptotic pathways, as well as pro-apoptotic members of the Bcl-2 gene family, Bak and Bax [53], have been shown to be up regulated in response to IFN. However, neither p53 nor Bcl-2 appear to be required for IFN mediated induction of apoptosis [53,56,57].

Although traditionally associated with the antiviral activity of IFNs, 2’-5’ OAS and RNaseL may also play a role in induction of apoptosis and antiproliferative responses through the inhibition of cellular protein synthesis by degradation of cellular RNA [58,59]. Furthermore, the antiviral protein, Protein Kinase R (PKR), acts to promote apoptosis in virus-infected cells [60]. Over expression of PKR results in toxic effects as well as increased susceptibility to induction of apoptosis [55]. 

Type I-IFN stimulation also leads to phosphorylation of the insulin receptor subunits 1 and 2 (IRS1 and IRS2), resulting in subsequent binding of the p85 regulatory subunit of phosphatidylinositol 3-kinase (PI3K) [61,62]. Protein Kinase C-δ (PKC-δ) is then activated and can act in the downstream regulation of apoptosis [63,64] (Figure 1)

The role of the PI3K/mTOR pathway in apoptosis remains unclear. PI3K has been shown to function as a cell survival factor as well an inducer of apoptosis [65]. In tumor cells, PI3K/mTOR is necessary for the induction of apoptosis after treatment with IFN-α [66]. In addition, PI3K has been shown to be involved in the regulation of cytokine expression. Activation of PI3K pathways, results in production of interferon-γ [67,68].

### 2.3. Anti-tumor

In addition to an antiproliferative or apoptotic effect, interferon treatment is known to also be capable of eliciting a direct cytotoxic effect on some tumor cells. One of the first references to this effect was in work done by Vilcek and others 25 years ago, in which he found that of five tumor lines tested, two, an adenocarcinoma line and a rhabdomyosarcoma line, were highly sensitive to either purified natural or recombinant interferon-gamma even at concentrations of 1 unit/mL with the effect being abrogated by the addition of a specific monoclonal anti-interferon gamma antibody [69]. Almost 10 years after this, Einhorn and colleagues observed a direct cytotoxic effect of interferon (IFN-α2b) on malignant cells from a patient with multiple myeloma. Interestingly, this effect was not observed with other malignant cells compared in the same study, namely cells from patients with B-cell lymphoma, chronic lymphocytic leukemia, hairy cell leukemia and chronic myelogenous leukemia raising the possibility that a direct cytotoxic effect of interferon in myeloma cells may be specific for this malignancy [70]. Worthy of mention here is the fact that two of the above-mentioned malignancies not undergoing a direct cytopathic effect by IFN-α treatment, Hairy cell leukemia and chronic myelogenous leukemia are two of the approved indications for IFN-α 2 in the United States, the first being approved for both IFN-α2a (Hoffman-La Roche^©^) and –α2b (Schering-Plough^©^) and having the distinction of being the first approved indication for both in 1986 whereas chronic myelogenous leukemia (CML) is an approved indication only of IFN-α2a. 

### 2.4. Indirect / Immunomodulatory effects of Interferon on cell growth and survival

Interferon treatment results in reduced production of basic fibroblast growth factor (bFGF) in human renal carcinoma cells [71], as well as a reduction in transcription and secretion of the vascular growth factor VEGF [72,73,74]. This could account for reduction in angiogenesis after IFN treatment of some tumors [75]. In addition, IFN has been shown to have a negative effect on hematopoiesis, which may account for some of the negative side effects of IFN therapy [76,77]. It has been shown that IFN-α can enhance the expression of the Epidermal Growth Factor Receptor (EGF-R) in some tumor cells (e.g. bladder cancer cell lines and KB cells) and that EGF can antagonize the apoptotic cell death induced by IFN-α and that IFN-α, in turn, can enhance the activity of EGF on certain cells [78,79,80].

Type-I and Type-II IFNs also play a role in T cell differentiation and B cell development, and may also aid in immune surveillance by increasing antigen processing and up regulating MHC-I expression, thus facilitating recognition by cytotoxic CD8+ T-cells. In addition, interferon-γ can also up regulate expression of MHC-II in antigen presenting cells, resulting in enhanced CD4+ T-cell response [81,82].

The presence of IFN may also result in the activation of moncytes and macrophages. Activated macrophages produce reactive oxygen species (ROS) and reactive nitrogen intermediates (RNI) which have cytotoxic effects on targeted cells. [83] In addition, activated monocytes produce cytokines, which initiate a Th1 response. 

Whether it is inhibition of proliferation, apoptosis, a direct cytotoxic effect or an immunomodulatory one, it should be noted that IFN’s ability to elicit these mechanisms varies considerably between cells of different lineage. The level of responsiveness to interferon may be related to genetic and/or signaling variances at different stages of the cell cycle. [84] There is also a variance based on cell type with some cells responding more strongly than others to the IFN used for treatment. 

## 3. Antitumor Activity of Interferon-Activated Monocytes *in Vitro*.

The immunomodulatory properties of IFNs include strong activation of monocytes/macrophages [85,86]. Activated monocytes are established to play a natural role against bacteria, viruses, and tumor cells [87,88,89,90,91,92,93,94]. Non IFN activators of macrophages might also act by inducing IFNs [85,95]. These include CpG [96,97] lipoplysaccaride (LPS),monoclonal antibodies (mAb) to surface molecules on monocytes [98], as well as dsRNA and other nucleic acids [99], transforming growth factor [100], cytokines and chemokines [101,102,103,104]. In the early antitumor studies of macrophages the degree of antitumor activity was substantial but did not reach eradicative levels but recent studies are more promising, reporting near-eradicative effects in some cases [89,99]. Importantly, the antitumor activity of activated monocytes appears to be relatively selective for tumor cells over normal cells. [89,105,106]. Also, since other leukocytes may exert antitumor activity, it is important that the effector cells in the reported monocyte studies were shown to be monocytes and not other contaminating cell types [89].

Some experimental clinical studies used IFN in combination with other cytokines or lymphocytes or macrophages to treat cancer. [87,105]. These adoptive innate immunotherapies have had encouraging success against some tumors and limited success with other tumors. The limited successes may indicate that more effective immunotherapies are needed. 

### Effect of IFN-activated human monocytes on some tumor cell lines

When 30-100 colony forming units of tumor cells were exposed to the combination of IFN-α plus purified monocytes, tumor colony formation was reduced by 20-fold (95%) compared with 2-fold inhibition by monocytes alone or 3-fold by IFN-α alone. It appears the reduction of both colony size and colony number supports the antiproliferative and cytocidal actions of the IFN and monocyte mixture. Similar results were obtained with both LOX melanoma and A549 lung tumors. Completely resistant to the eradicative effects of human monocytes and IFN is the human glioma SNB-19. Of note is the fact that the minimal effective concentration of IFNs needed to activate antitumor activity of monocytes approach the very low concentrations that activate antiviral activity (10^8.3^antiviral units/mg). Interestingly, no inhibition was observed when the human diploid cell lines, WI38, FS4, and MRC5 were assayed as above [107].

Direct contact between the human monocytes and the tumor cells was required for this inhibitory effect as opposed to said effect being the result of soluble cytokines alone [89,99,105,107,108,109,110]. Consequently, methods to optimize direct contact *in vivo* might be essential for eradication *in vivo*. In a small study of tumor patients treated locally or distally with IFNγ-activated monocytes [111,112] reduction of ascites tumors in two of seven patients occurred only when the monocytes were administered locally (i.p.) [105,106,113]. In these patients, low-grade fever and laboratory changes in fibrin accompanied infusion of human monocytes activated with IFNγ (10^8^ units) [105,106,114]. Toxicity of the activated monocytes for normal cells may not be a limiting problem, as the former do not appear to be cytotoxic for normal diploid cells or in mice [89,98,109,111,115,116] and these side effects were tolerated by patients. Furthermore, direct local therapy of tumors may minimize systemic side effects.

Of medical importance would be a demonstration of this strong antitumor activity in animal models and whether established tumors can be eradicated [115,117]. Also important would be identification of the mechanisms responsible for the higher sensitivity of many tumor cells over diploid cells [109]. Thus, because of its potency and relative specificity, the IFN activated monocyte may be a candidate for therapy of human tumors such as localized skin and brain tumors as well as residual tumors at the site of excision. An additional advantage may be that this local therapy of tumors may minimize systemic side effects.

## 4. Clinical Applications

The only interferons licensed for anti-tumor applications are IFN-α2a (Roferon-A, Hoffman-La Roche, Nutley, NJ) and IFN-α2b (Intron-A®, Schering-Plough, Kenilworth, NJ) (Table 1).

### 4.1. Chronic Myelogenous Leukemia

In 1987, Gutterman, *et al*. first addressed the use of IFN-α in the treatment of Chronic Myelogenous Leukemia (CML) and observed a 71% positive response rate in 51 patients treated with doses of 3 to 9 million international units (MIU) daily but it was not established as the standard of care for the disease until the early 1990s when it was determined to be better than standard chemotherapy by several randomized controlled trials [118]. Interestingly, CML was the first human malignancy in which a consistent chromosomal abnormality was identified. This is a reciprocal translocation between chromosome 9 and 22 (BCR-ABL translocation) resulting in the formation of what is known as the Philadelphia + (Ph+) chromosome. Since approximately 95% of patients with CML have this abnormality, its presence is a sensitive test for the disease although it is not specific enough to diagnose it as it can also be found in patients with acute lymphoblastic leukemia [119]. Suppression of the Ph chromosome accompanied by complete hematological repression has been seen with IFN-α administration [120], but as in some of the other malignancies mentioned above, higher response rates have been observed in CML when IFN-α is used in combination with chemotherapy (e.g. cytosine arabinoside) [121]. Although pegylated IFN-α2a (Pegasys) is not licensed for the treatment, recent randomized trials revealed that it showed significantly greater complete hematologic as well as cytogenetic responses than those observed when unpegylated IFN-α was used in the treatment of CML patients. Imatinib (Gleevec), which is also licensed for the treatment of CML, and IFN-α are the two most active forms of non-transplant therapy and have been shown, *in vitro*, to have additive or synergistic antiproliferative effects using cells positive for the above-mentioned chromosomal abnormality (BCR-ABL) and in colony-forming assays using samples from CML-positive patients. [121] Discontinuation of Gleevec has been shown to result in very high relapse rates which might be the result of a resistance to the drug by CML stem cells. Interestingly, patients initially receiving IFN-α who then were switched to Gleevec treatment demonstrated long term remission at a high rate. It has been proposed that this pre-treatment with IFN-α results in the CML stem cells becoming sensitized to Gleevec [122]. A number of clinical trials are in place designed to compare either combination IFN-α/Imatinib or combination PEG-IFN-α/Imatinib therapies (www.clinicaltrials.gov).

### 4.2. Follicular Lymphoma

This malignancy is a slow-growing, low grade (indolent) Non-Hodgkin’s lymphoma and its incidence has increased over the past twenty years. Extensive studies have been conducted on the use of IFN-α2 both in conjunction with chemotherapy and/or as maintenance therapy after remission has been accomplished but the results of these studies have been mixed. Some trials which used IFN have shown both prolonged Progression-Free Survivals (PFS) as well as Overall Survivals (OS) whereas in others, there was no demonstrable survival improvement [123,124]. The safety and efficacy of Intron-A® (IFN-α2b) in combination with the chemotherapeutic cocktail CHVP (cyclophosphamide, doxorubicin, vindesine and prednisone) was assessed by its manufacturer (Schering-Plough) in patients with Stage III/IV follicular Non-Hodgkin’s Lymphoma with one study group receiving both and the other receiving IFN-α2b alone. Progression-free survival among the patients receiving combination therapy was significantly greater (2.9 *vs.* 1.5 years) compared to the patients who received IFN monotherapy. The median survival after a 6.1 year follow-up was 5.5 years in patients treated with CHVP alone while that of patients treated with both CHVP and Intron-A® has not yet been reached (Intron-A® package insert). The use of IFN-α2b in conjunction with chemotherapy in patients with follicular lymphoma was approved by the FDA in November of 1997. Comparison of combination chemotherapy plus IFN-α and combination chemotherapy and Rituximab (a monoclonal antibody also licensed for Follicular Lymphoma) is the subject of an ongoing clinical trial.

### 4.3. Malignant Melanoma

Safety and efficacy of Intron-A® was evaluated as an adjuvant to surgical treatment in patients with melanoma who were disease free (post surgery) but still considered to be at high risk for systemic recurrence. In a randomized control trial comparing high-dose IFN-α2b to patient observation (N = 280), 143 patients received 20 MIU/m^2^ of Intron-A® (I.V., 5 times per week x 4 weeks) followed by a maintenance dose of 10 MIU/m^2 ^(S.C, 3 times per week x 48 weeks) while the remaining patients (N = 137) underwent observation for that time period. Both relapse-free and overall survival rates were increased in the patients receiving Intron-A®. (Intron-A® Package Insert) Conflicting results from additional trials has led to controversy about the use of IFN-α2b as an adjuvant therapy in the treatment of malignant melanoma. Nevertheless, the number of clinical trials which demonstrate relapse-free survival rates with the use of high-dose IFN-α2b in this disease still supports this treatment option today. In addition, high-risk patients who have chosen to forego initial IFN-α therapy receive some benefit from it should they present with a resectable recurrence of the malignancy after either observation or another form of initial therapy [125]. Clinical trials are ongoing comparing the therapeutic efficacy of IFN-α or other cytokines (e.g., interleukin-2 and interleukin-12) with or without chemotherapeutics (e.g., vinblastine, cisplatin) in treating malignant melanoma in addition to those in which biologics (e.g. IFN-α, aldesleukin, IL-2) are administered subsequent to chemotherapy. (www.clinicaltrials.gov). 

### 4.4. Hairy Cell Leukemia

Although Interferon (IFN) was first described as an antiviral agent both IFN-α2a (Roferon-A®, Hoffman-La Roche) and IFN-α2b (Intron-A®, Schering-Plough) were licensed by the U.S. FDA for the treatment of the B-cell neoplasm known as Hairy Cell Leukemia (HCL). At that time, a high degree of efficacy was observed in its use against the disease as evidenced by reduction in cytopenia and clearing of the malignant “hairy cells” from the blood, the presence of both being classic symptoms of the disease [126]. Today, however, IFN is not the standard of care in the treatment of HCL as better results have been seen using purine analogues (cladribine, pentostatin, e.g.) instead [127]. Interestingly, cladribine is also used in the treatment of multiple sclerosis, the only application for which IFN-β is licensed.

### 4.5. AIDS-Related Kaposi’s Sarcoma

In November 1988, IFN-α2a and –α2b were licensed for the treatment of AIDS-related Kaposi’s Sarcoma (KS). In both cases, likelihood of response to the IFN therapy is greater in patients not having systemic symptoms, who have limited lymphadenopathy and who have relatively intact immune systems. (Intron-A® Package Insert). Dosing trials with Roferon-A using an escalating regimen of 3 MIU, 9 MIU, and 18 MIU each daily for three days followed by 36 MIU daily were performed. These showed that the 36 MIU dose as well as the escalating regimen provided the best response. Lower doses showed poor tumor regression and doses higher than 36 MIU resulted in unacceptable toxicity and in 2003 Hoffman-La Roche requested of the FDA that the Kaposi’s Sarcoma indication, 18 and 36 MIU dose vials be removed from their license. (Roferon-A® Package Insert). Lower doses, however, did prove effective when used in conjunction with anti-retroviral therapy which had the added benefit of minimizing the interferon-related toxicity. As a single agent, doses of 30 MIU or more were required to attain tumor regression. A randomized control trial was conducted on patients with AIDS-related Kaposi’s Sarcoma in which one group received a low dose (1 MIU) or an intermediate dose (10 MIU) of IFN-α2b once daily with twice daily doses of the reverse transcriptase inhibitor didanosine (Videx). The response rate for the low dose was 40% and that for the intermediate dose was 55% and there was no significant difference in survival rate between the two groups. [128] Given the higher toxicity observed with the intermediate dose, use of the low (1 MIU) dose may be preferable. The standard of care for AIDS-related KS is HAART (highly active antiretroviral therapy) in which health care providers prescribe a “cocktail” of antiretroviral drugs (currently the FDA has approved at least 28 of these) and they fall into three major categories: reverse transcriptase inhibitors, fusion inhibitors and protease inhibitors. These new drugs have appeared to surpass IFN-α in the treatment of this disease even though it is still one of the more active agents used to treat it. Both recombinant IFN-α2a and IFN-α2b as well as lymphoblastoid IFN-α are being used in clinical trials in conjunction with the nucleoside analogue zidovudine (AZT), known to be active against HIV-1, in the treatment of patients with AIDS-related Kaposi’s Sarcoma (www.clinicaltrials.gov). 

**Table 1:** Interferon-αs licensed by the U.S.F.D.A. which have anti-tumor applications (highlighted in red). *The market and sale of Roferon-A in the United States was discontinued in September 2007. This action was NOT due to the safety or efficacy of the product.

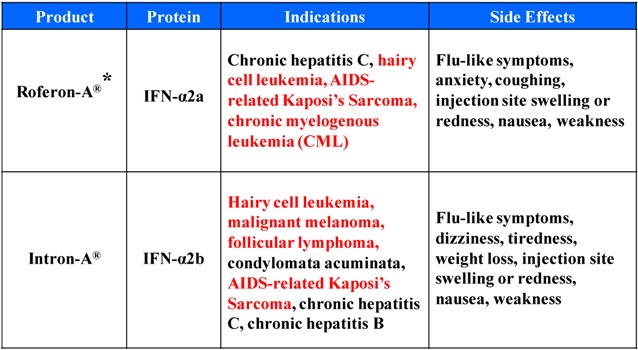

Even though it is no longer licensed in the United States, Wellferon (IFN-alpha n1), which is a natural IFN, was licensed for the treatment of Juvenile Laryngeal Papillomatosis (JLP). The manufactuer (then Glaxo-Wellcome) withdrew the license for marketing purposes, not because the interferon showed no efficacy.

## 5. Pharmacokinetics of IFN-αs Licensed by the U.S. FDA

### 5.1. IFN-α2a

Pharmacokinetics of a single intramuscular dose of IFN-α2a (3-198 MIU) are linear in both healthy volunteers and patients with disseminated cancer with peak serum concentrations ranging from 1,500 to 2,580 pg/mL after a single intramuscular (IM) administration of 36 MIU (mean time to peak 3.8 hrs) and a range of 1,250 to 2,320 pg/mL after subcutaneous (SC) administration (mean time to peak 7.3 hours). It should also be noted that a wide intrasubject variation in serum concentration has been observed in the two groups. Bioavailability after intramuscular administration ranges from 80-83% with total body clearance being between 2.14 and 3.62 mL/min per kg. [129] No change was seen in either distribution or elimination over a dosing period of 28 days regardless of whether the IFN was administered once daily (1-54 MIU), twice daily (0.5-36 MIU) or three times weekly (1-136 MIU). (Roferon-A® Package Insert, www.medsafe.govt)

### 5.2. IFN-α2b

Healthy volunteers (N = 12) were administered 5 MIU/m^2^ of IFN-α2b either subcutaneously (SC), intramuscularly (IM) or as a 30 minute intravenous (IV) infusion. Mean serum concentrations following SC and IM dosages were comparable with maximum concentrations observed 3-12 hours post administration and the elimination half-life being 2-3 hours becoming undetectable at 16 hours. Serum concentrations peaked at the end of the infusion for the IV administration and became undetectable at 4 hours after the infusion. There were no detectable levels observed in urine samples after any of the administration routes (Intron-A® package insert).

## 6. Antibodies to Interferon-α

It is known that IFN-α therapy can result in the development of both neutralizing and non-neutralizing IFN-α antibodies. After IFN-α2b therapy, serum neutralizing antibodies were detected in 0% (0/90) of Hairy Cell Leukemia patients, 4% (1/24) of patients with AIDS-related Kaposi’s Sarcoma and <3% of patients with other malignancies (Intron-A® package insert). Although the manufacturer of IFN-α2b (Schering-Plough) reports that the presence of these antibodies did not appear to negatively impact safety of efficacy in their studies, they have the ability to reduce the latter thereby resulting in a relapse or refractory disease. More than 10 years ago, Őberg, *et al*. examined the clinical significance of IFN-α antibodies in 327 patients with solid tumors, 215 of which received IFN-α2b (Intron-A®) and 29 received IFN-α2a (Roferon-A®). 17% of those receiving Intron-A® developed neutralizing antibodies but high-titers (>800 neutralizing units/mL) were seen in only 4% of the patients. The incidence of neutralizing antibodies developed in patients receiving Roferon-A® was greater with 38% being positive in a neutralization assay and 28% having high-titer neutralizing antibodies. Of the two groups, there were a total of 17 patients (9 on IFN-α2b and 8 on IFN-α2a) with high-titer antibodies and all but five of these had loss of antitumor response as evidenced by higher levels of tumor markers as well as tumor progression [130]. Reports of the clinical significance of the development of neutralizing antibodies in patients with Hairy Cell Leukemia, Chronic Myelogenous Leukemia and Renal Cell Carcinoma as well as carcinoid tumors, show that 63% of those who developed neutralizing antibodies to IFN-α experienced relapse or develop resistance to the interferon used for initial treatment [131]. In the case of long term therapy with IFN-α2a for Hairy Cell Leukemia, however, antibody presence is transient with 100% of patients studied having non-neutralizing antibodies becoming antibody-negative after a median of 14.5 months while 30% of those having neutralizing antibodies becoming antibody-negative [132].

## 7. Conclusions

Over the past 25 years, a number of clinical applications of IFN-α have been licensed by many regulatory agencies around the globe for a variety of cancers. However, much still needs to be learned about the mechanism(s) of IFN’s antitumor activity, both its direct roles, e.g. inhibition of cell growth and apoptosis and its indirect activity on the immune cells which activates them to kill the tumor cells. In addition, there is also a need to understand the mechanism of IFN’s toxicity so that it can be reduced in its clinical applications and thus making it more suitable as an anti-tumor agent either alone or in combination with other antitumor agents.
